# Theory of synergistic effects: Hill-type response surfaces as ‘null-interaction’ models for mixtures

**DOI:** 10.1186/s12976-017-0060-y

**Published:** 2017-08-02

**Authors:** Michael Schindler

**Affiliations:** Institute of Theoretical Chemistry, Ruhr-University Bochum, Universitätsstr., Bochum, Germany

**Keywords:** Synergistic effects, Hill response surface, Loewe additivity, Bliss independence, Colby’s formula

## Abstract

**Background:**

The classification of effects caused by mixtures of agents as synergistic, antagonistic or additive depends critically on the reference model of ‘null interaction’. Two main approaches are currently in use, the Additive Dose (ADM) or concentration addition (CA) and the Multiplicative Survival (MSM) or independent action (IA) models. We compare several response surface models to a newly developed Hill response surface, obtained by solving a logistic partial differential equation (PDE). Assuming that a mixture of chemicals with individual Hill-type dose-response curves can be described by an n-dimensional logistic function, Hill’s differential equation for pure agents is replaced by a PDE for mixtures whose solution provides Hill surfaces as ’null-interaction’ models and relies neither on Bliss independence or Loewe additivity nor uses Chou’s unified general theory.

**Methods:**

An n-dimensional logistic PDE decribing the Hill-type response of n-component mixtures is solved. Appropriate boundary conditions ensure the correct asymptotic behaviour. Mathematica 11 (Wolfram, Mathematica Version 11.0, 2016) is used for the mathematics and graphics presented in this article.

**Results:**

The Hill response surface ansatz can be applied to mixtures of compounds with arbitrary Hill parameters. Restrictions which are required when deriving analytical expressions for response surfaces from other principles, are unnecessary. Many approaches based on Loewe additivity turn out be special cases of the Hill approach whose increased flexibility permits a better description of ‘null-effect’ responses. Missing sham-compliance of Bliss IA, known as Colby’s model in agrochemistry, leads to incompatibility with the Hill surface ansatz. Examples of binary and ternary mixtures illustrate the differences between the approaches. For Hill-slopes close to one and doses below the half-maximum effect doses MSM (Colby, Bliss, Finney, Abbott) predicts synergistic effects where the Hill model indicates ‘null-interaction’. These differences increase considerably with increasing steepness of the individual dose-response curves.

**Conclusion:**

The Hill response surface ansatz contains the Loewe additivity concept as a special case and is incompatible with Bliss independent action. Hence, when synergistic effects are claimed, those dose combinations deserve special attention where the differences between independent action approaches and Hill estimations are large.

## Background

Quantifying the effect of an active substance upon application to its target organism is a central topic in life sciences. The description and prediction of dose-response curves of pure compounds and estimating the combined effect of simultaneous administration of several active ingredients (a.i.s) is a field of active research in pharmacology, anesthesiology, toxicology, environmental science, and agrochemistry. The detection of virtually any kind of interaction between drugs is, e.g., of utmost importance for risk assessment in the registration process of chemicals in general. While synergistic adverse effects present a challenge to say the least, synergistic effects in drug administration are desirable as they might be used to reach the same goal with less effort. Disregarding the steadily growing area of possible interactions of chemicals with their environment, this article concentrates on interactions between a.i.s in the life-sciences. The terms ‘agent’, ‘drug’, ‘chemical’, and ‘a.i.’ are used interchangeably.

In 1989, Berenbaum [1] summarized the state of the art describing synergistic effects in pharmacology. Since then, numerous attempts to quantify non-additive effects have been published, mainly in the fields of chemotherapy [2], anesthesiology [3], toxicology [4], physiology [5], environmental science [6, 7], and pharmacology [8], to quote only a few reviews.

Generally, biological effects of an a.i. follow a dose-response relation, starting from a no-effect level (NOEL) and ending at the maximum effect corresponding to a saturation dose. In order to quantitatively describe interactions between substances, it is necessary to define a reference behaviour, namely the response of a system of compounds acting independently. Deviations from this ideal reference can then be classified as synergistic or antagonistic, being aware of the fact that these deviations can be caused by a multitude of physico-chemical and biochemical effects, especially in whole organisms (for a recent study see e.g. [9]).

Whenever the effect observed after applying a mixture exceeds the expectation, the action of the agents is called synergistic, and if it is smaller than expected it is antagonistic. This seemingly simple definition is not simple at all, as there is an ongoing debate since the beginning of the last century on how to correctly define this reference of ‘no interaction’. In the literature, synergy is defined either phenomenologically or based on assumptions on the modes of action of the a.i.s involved. The observed effects like zero-interaction, synergism or antagonism, are often quantified by calculating interaction- [10] or combination-indices [11], however, often based on differing definitions of additivity [2, 4].

Mainly two types of reference models are currently in use [12, 13]. One class of models, the Additive Dose Model (ADM) pioneered by Loewe [14], assumes an additive behaviour of doses, similar modes of action and consequently parallel dose-response curves with identical slopes. Its basic concept is that of equipotent doses, which means that an arbitrary dose of a.i. A can be replaced by an isoeffective dose of a.i. B. The terms ‘effect summation’, ‘dose addition’, and ‘concentration addition (CA)’ are used in this context. As the joint action of the a.i.s is not independent in this model, the term ‘mutually exclusive’ [11] is more appropriate here. Implicitly the model assumes that the maximum possible effect achievable by the mixture is that of its most potent partner. A generalized concentration addition (GCA) model [6] extended the original ADM approach [10, 14–16] to mixtures with partially overlapping agonists.

The other class of models, called Multiplicative Survival Models (MSM), assumes that the effects caused by the two a.i.s are mutually non-exclusive and originate from different modes of action, and hence the asymptotically achievable effect is the sum of the individually possible ones. In agrochemical research, it is associated with the names of Colby [17] and Limpel [18], while in toxicology these models of independent action (IA) have been described by Bliss [19] and Finney [20] and in entomology by Abbott [21].

Chou’s widely used combination indices [11] cover both classes, his formula for the mutually exclusive combination is of ADM type while his description of mutually non-exclusive mixtures is MSM like and reduces to Colby’s formula for reaction orders of one. The approach presented here relies neither on Bliss independence or Loewe additivity nor uses Chou’s unified general theory. It focuses on logistic (Hill) response surfaces as ‘null-effect’ models. For mixtures of n partners they result from solving an n-dimensional partial differential equation (PDE). Appropriate boundary conditions guarantee that the expressions describing the Hill-surfaces are asymptotically correct, i.e., lead to Hill’s well-known dose-response curves in the one-dimensional case of pure a.i.s. In addition, the solutions are sham-compliant, meaning that a mixture consisting of combinations of a drug with itself shows no synergistic effect.

Sigmoid dose-response curves like Hill’s equation [22, 23] are solutions of a class of ordinary differential equations (ODEs) which was originally used to describe population dynamics [24]. Examples of the phenomena described by this type of functions are titration curves in chemistry, dose-response curves in enzyme kinetics, and predator-prey models in biology.

Following a short recapitulation of the ODE leading to Hill-type dose-response curves for pure compounds, Hill response surfaces for binary and n-component mixtures are introduced as solutions of the corresponding PDEs. In the subsequent sections the properties of Hill surfaces are compared to other response surface models and applied to some literature examples, namely binary and ternary mixtures from various fields of life-science. A summary of our findings will be given in the last section.

## Methods

While Hill’s equation can be obtained by solving a first-order ODE, the logistic differential equation, its n-dimensional generalization results from solving a semilinear PDE with the appropriate boundary conditions. These are the requirements that in the limit of one dimension the original Hill equation results, and that the solution of the PDE is sham-compliant, meaning that an artificial partitioning of the one-dimensional problem into an n-dimensional one does not change the results.

Here an analytical expression of the n-dimensional extension of Hill’s equation is provided. Verification is done by substituting the solution into the PDE by using Mathematica 11 [25]. Checking the fulfillment of the boundary conditions is achieved by performing the corresponding limiting processes.

## Results

### Logistic functions and the Hill response surface

Let A and B be active ingredients with dose-response curves *a*(*x*) and *b*(*y*) depending on the variables *x* and *y* and let U be a combination of A and B with the dose-response surface *u*(*x,y*). Let us further assume that we can describe the effect *z* (being *a*(*x*), *b*(*y*) or *u*(*x,y*)), by a logistic function. Then *z* is characterized by 4 parameters, the minimum and maximum responses *z*_*min*_ and *z*_*max*_, the position of the inflection point and a slope parameter, i.e., the slope at the inflection point of the curve. All effects are limited by the no-effect- and the full-effect levels, 0≤*z*_*min*_≤*z*≤*z*_*max*_≤1.

The differential equations for the one- and two-dimensional cases, describing the variation of the effects *a*(*x*), *b*(*y*) and *u*(*x,y*) with the variation of *x* and *y*, are

1 $$\begin{array}{@{}rcl@{}} \frac{da(x)}{dx}= \alpha a(x)\left(1-\frac{a(x)}{a_{max}}\right) \; \ \ \ \ \; \frac{db(y)}{dy}= \beta b(y) \left(1-\frac{b(y)}{b_{max}}\right)  \end{array} $$

2 $$\begin{array}{@{}rcl@{}} u_{x} + u_{y} = \gamma (x,y) u(x,y) \left(1 - \frac{u(x,y)}{u_{max}(x,y)}\right)  \end{array} $$

where *u*_*x*_=*∂* *u*(*x,y*)/*∂* *x* and *u*_*y*_=*∂* *u*(*x,y*)/*∂* *y* denote the partial derivatives of *u*, and *α*, *β* and *a*_*max*_, *b*_*max*_ are constants. In Eq. 2, *γ* and *u*_*max*_ are functions of *x* and *y*.

Denoting by *x*_50_ and *y*_50_ the positions of the half-maximum effects *a*(*x*_50_)=*a*_*max*_/2 and *b*(*y*_50_)=*b*_*max*_/2 the final forms of *a*(*x*) and *b*(*y*) are the logistic functions

3 $$\begin{array}{@{}rcl@{}} a(x) = \frac{a_{max} e^{\alpha \Delta x}}{1+ e^{\alpha \Delta x}} = \frac{a_{max}}{1+ e^{-\alpha \Delta x}} \; \ \ \ \ \; b(y) = \frac{b_{max} e^{\beta \Delta y}}{1+ e^{\beta \Delta y}}=\frac{b_{max}}{1+ e^{-\beta \Delta y}}  \end{array} $$

with *Δ* *x*=*x*−*x*_50_, *Δ* *y*=*y*−*y*_50_ and *α* and *β* being the slopes at the inflection points *x*_50_ and *y*_50_. They show an exponentially increasing effect at low doses, becoming linear close to *x*=*x*_50_ and *y*=*y*_50_ and finally an exponentially decreasing growth until the limiting effect *z*_*max*_ is reached. *a*(*x*) and *b*(*y*) are intimately connected to Hill’s equation, providing a relation between the effect *E* and the dose *C*.

4 $$\begin{array}{@{}rcl@{}} E=E_{0}+\frac{E_{max} C^{\alpha}}{C^{\alpha}+{EC}_{50}^{\alpha}} =E_{0}+\frac{E_{max}}{1+\left(\frac{{EC}_{50}}{C}\right)^{\alpha}}  \end{array} $$

Actually, *x* and *y* are the natural logarithms of doses with −*∞*≤*x,y*≤*∞*, whereas the doses themselves (i.e., *e* ^*x*^ and *e* ^*y*^) are ≥0. Hence, we can identify the effects *E*_0_, *E*_*max*_ and the shape parameter *α* with *z*_*min*_, *z*_*max*_ and *α* or *β*, and the doses *C* and *EC*_50_ with *e* ^*x*^ and $\phantom {\dot {i}\!}e^{x_{50}}$ or *e* ^*y*^ and $\phantom {\dot {i}\!}e^{y_{50}}$ of Eq. 3, meaning that Hill’s equation is the solution of a logistic ODE, subject to the appropriate boundary conditions.

Our approach to handle mixtures is completely analogous to that used for the pure compounds. The solution of the PDE describing the effect *u*(*x,y*) of a binary mixture (Eq. 2) is the logistic Hill-surface as the response surface of ‘null-interaction’.

5 $$\begin{array}{@{}rcl@{}} u_{Hill}(x,y) & = & \frac{u_{max}(x,y)}{1+ \left[e^{\Delta {x}} + e^{\Delta {y}}\right]^{-\gamma(x,y)}}  \end{array} $$

The boundary condition

$$\begin{array}{@{}rcl@{}} u(x,-\infty)=a(x) \; \wedge \; u(-\infty,y)=b(y)  \end{array} $$

that in the limit of one vanishing agent the response due to the second one has to result, and the so-called sham-compliance requirement have to be fulfilled: If a dose *d* of one a.i. is artificially split into two contributions *n d* and (1−*n*)*d* with 0≤*n*≤1, the response due to this ’mixture’ has to be identical to the response of the pure compound, i.e., *a*(*d*)=*u*(*n*×*d*,(1−*n*)×*d*), irrespective of any possible interaction between different a.i.s. In our formalism a dose *d* is equal to *e* ^*x*^. Hence, sham partitioning means setting *e* ^*x*^=*n e* ^*x*^+(1−*n*)*e* ^*x*^, leading to the requirement *u*(*x*+ ln*n,x*+ ln(1−*n*))=*a*(*x*).

These conditions are satisfied by the ansatz

6 $$ \begin{aligned} \gamma(x,y) & = \frac{\alpha e^{\Delta x} + \beta e^{\Delta y}}{e^{\Delta x}+e^{\Delta y}} \\ u_{max}(x,y) & = \frac{a_{max} e^{\Delta x} + b_{max} e^{\Delta y}}{e^{\Delta x}+e^{\Delta y}} \end{aligned}   $$

which provides smooth transitions between *α* and *β* and between *a*_*max*_ and *b*_*max*_.

To facilitate a comparison with literature expressions for response surfaces, *u*_*Hill*_ can be re-written by substituting the doses *d*_*a*_ and *d*_*b*_ for *e* ^*x*^ and *e* ^*y*^ and using the doses scaled by their median effects $\phantom {\dot {i}\!}m_{a}=d_{a}/d_{a_{50}}= e^{\Delta x} $ and $\phantom {\dot {i}\!}m_{b} =d_{b}/d_{b_{50}}= e^{\Delta y}$. Then the ‘null-interaction’ reference response surface is

7 $$\begin{array}{@{}rcl@{}} u_{Hill} = u_{max}(m_{a},m_{b}) \times \frac{\left(m_{a} +m_{b}\right)^{\gamma(m_{a},m_{b})}}{1+ \left(m_{a} +m_{b}\right)^{\gamma(m_{a},m_{b})}}  \end{array} $$

with

8 $$ \begin{aligned} u_{max} &= \frac{a_{max}m_{a}+b_{max}m_{b}}{m_{a}+m_{b}}\\ \gamma &= \frac{\alpha m_{a} + \beta m_{b}}{m_{a}+m_{b}} \end{aligned}   $$

As the ansatz presented here uses the known dose-response curves of the pure mixture partners to unambiguously define the ‘null-interaction’ surface for the binary mixture, there is no obvious way to introduce interaction parameters (and functions) which account for deviations from this reference [26–28]. At present the only way to detect synergism or antagonism is the direct comparison of experimental data with the Hill response surface, either visually or by statistical means like the root-mean squared error of prediction (RMSE).

A way out of this dilemma might be to obtain the necessary parameters directly from fitting the experimental mixture data to a logistic surface which could then be compared to the ‘null-interaction’ model. However, the functional form of *u*_*Hill*_ is not flexible enough for this purpose and some sort of perturbation theory might be more appropriate to handle deviations from the Hill surface.

### The logistic PDE for mixtures of n components

The extension of the Hill formalism to mixtures of n agents *A*_*i*_ is straightforward. The corresponding semilinear logistic PDE [29] is

9 $$\begin{array}{@{}rcl@{}} \sum\limits_{i=1}^{n}{u_{x_{i}}}= \gamma(\vec x) u(\vec x) \left(1-\frac{u(\vec x)}{u_{max}(\vec x)}\right)  \end{array} $$

where $\vec x = (x_{1},\ldots,x_{i},\ldots,x_{n})$ and $u_{x_{i}}=\partial u(\vec x)/\partial x_{i}$. Its solution

10 $$\begin{array}{@{}rcl@{}} u(\vec x) = u_{max}(\vec x) \frac{\left[\sum\limits_{i=1}^{n} {e^{\Delta {x_{i}}}}\right]^{\gamma(\vec x)}}{1+ \left[\sum\limits_{i=1}^{n}{e^{\Delta {x_{i}}}}\right]^{\gamma(\vec x)}}  \end{array} $$

with

$$\begin{array}{@{}rcl@{}} \gamma(\vec x) = \frac{\sum\limits_{i=1,n}{\alpha_{i} e^{\Delta x_{i}}}}{\sum\limits_{i=1,n}{e^{\Delta x_{i}}}} \; \ \ \ \ \; u_{max}(\vec x) = \frac{\sum\limits_{i=1,n}{a_{{max}_{i}} e^{\Delta x_{i}}}}{\sum\limits_{i=1,n}{e^{\Delta x_{i}}}}  \end{array} $$

describes an n-dimensional logistic surface. It satisfies the boundary conditions,

$$\begin{array}{@{}rcl@{}} u(-\infty,\ldots,x_{i},\ldots,-\infty)=a_{i}(x_{i})  \end{array} $$

and the sham compliance condition, that any partition of the dose of a pure drug into various artificial new drugs must cause the same effect as the pure drug alone.

As an example, *u*_*Hill*_ for a ternary mixture of A, B and C satisfying the sham condition *a*(*d*)=*u*((*n*−*m*)×*d*,(1−*n*)×*d,m*×*d*) with 0≤*n*+*m*≤1, is

$$\begin{array}{@{}rcl@{}} u_{Hill} & = & u_{max} \times \frac{\left[m_{a} +m_{b}+m_{c}\right]^{\gamma}}{1+ \left[m_{a} +m_{b}+m_{b}\right]^{\gamma}}  \end{array} $$

where in a self-explaining notation

$$\begin{array}{@{}rcl@{}} u_{max} & = & \frac{a_{max}m_{a}+b_{max}m_{b}+c_{max}m_{c}}{m_{a}+m_{b}+m_{c}}\\ \gamma & = & \frac{\alpha m_{a} + \beta m_{b}+\delta m_{c}}{m_{a}+m_{b}+m_{c}}  \end{array} $$

## Discussion

From a theoretical point of view it is an advantage of the Hill surface approach that it does not rest on assumptions on maximum effects or restrictions on specific parameter combinations of the mixture components. This distinguishes our approach from other response surface models as will be outlined in the next section. In practical applications, however, these differences become significant only if steep dose-response curves and/or strongly differing maximum-effect parameters of the individual agents are involved.

### A Comparison of empirical response surface models

Many of the response surface models are based on two synergy approaches, the Bliss independence [19, 20] and Loewe additivity [14] models. The characteristics of some of them are listed in Table 1. There their functional forms describing the mixture effects are classified by their assumptions on maximum effects and slopes and by analyzing whether they are asymptotically correct and sham compliant. Apparently only a few authors [26, 28, 30] use the freedom to define the shape function $\gamma (\vec x)$ and the maximum effect function $u_{max}(\vec x)$ (Eq. 6).

**Table 1:** Characteristics of theoretical models describing mixtures

		Assumptions		Properties	
Approach	Eq.	*u*_*max*_	Slopes *γ*	Pure a.i. limit	Sham compliance
Bliss / Colby	13	Unequal	*γ*_*a*_≠*γ*_*b*_	Ok	No
Loewe / CA	14	Equal	*γ*_*a*_=*γ*_*b*_	Ok	Yes
GCA	18	Unequal	*γ*=1	Ok	Yes
*Chou*_*exc*_	19	Equal	*γ*_*a*_=*γ*_*b*_	Ok	Yes
*Chou*_*nonex*_	20	Equal	*γ*_*a*_=*γ*_*b*_	Ok	No
Greco	17	Equal	*γ*_*a*_≠*γ*_*b*_^a^	(Ok)^b^	(Yes)^b^
Minto / Fidler	21	Variable	Variable	(Ok)^c^	(Yes)^c^
Hill	5, 7	Variable	Variable	Ok	Yes

^a^analytical expression for the effect only for identical slopes

^b^for *α*=0

^c^if the polynomial expansions are truncated after the linear terms

Bliss independent joint action (IA) [3] can be formulated in terms of fractions of possible response unaffected *fu* or fractions affected *fa*, with *fa + fu*=1. For a binary mixture *Fu*_*ab*_=*fu*_*a*_×*fu*_*b*_ and *Fa*_*ab*_=1−*Fu*_*ab*_. Hence

11 $$\begin{array}{@{}rcl@{}} {Fa}_{ab} = 1 - (1-{fa}_{a})\times (1-{fa}_{b}) = {fa}_{a} + {fa}_{b} - {fa}_{a} \times {fa}_{b} \end{array} $$

It is related to the expression for the probability *P*(*A*∪*B*) of an event A or B if the basic events with probabilities *P*(*A*) and *P*(*B*) are independent. In crop science it is known as MSM [13] or Colby [17] model and is widely used to classify mixture effects [31]. Recently Colby’s formula has been extended to multi-compound mixtures [32]. As the IA ansatz is not sham-compliant it is not compatible with the Hill approach.

12 $$\begin{array}{@{}rcl@{}} u_{Colby}(x,y) &=& u_{Bliss}(x,y) = a(x)+ b(y)-a(x) \times b(y) \end{array} $$

13 $$\begin{array}{@{}rcl@{}} & =& \frac{a_{max}}{1+m_{a}^{-\alpha}}+\frac{b_{max}}{1+m_{b}^{-\beta}}-\frac{a_{max}b_{max}}{\left(1+m_{a}^{-\alpha}\right)\left(1+m_{b}^{-\beta}\right)}  \end{array} $$

The ADM [13] or CA model of mutually exclusive action [1, 14] for two noninteracting isoactive drugs A and B is

14 $$\begin{array}{@{}rcl@{}} 1 = \frac{m_{a}}{{f_{a}}^{-1}} + \frac{m_{b}}{{f_{b}}^{-1}}  \end{array} $$

where *f*_*x*_^−1^ is the dose or concentration of compound x that causes the specified effect. If the agents are acting according to Hill dose-response functions with slopes *γ*_*a*_ and *γ*_*b*_, it is given by

15 $$\begin{array}{@{}rcl@{}} 1 = \frac{m_{a}}{\left(\frac{u}{a_{max}-u}\right)^{1/\gamma_{a}}} + \frac{m_{b}}{\left(\frac{u}{b_{max}-u}\right)^{1/\gamma_{b}}}  \end{array} $$

Adapting Berenbaum’s approach, Greco derived a model for two-agent combined action by adding an interaction term, parameterized by a factor *α*. Assuming that the Hill-type dose-response curves of A and B differ only in the slope parameters, he gets [33]

16 $$\begin{array}{@{}rcl@{}} 1 = \frac{m_{a}}{\left(\frac{u}{u_{max}-u}\right)^{1/\gamma_{a}}} + \frac{m_{b}}{\left(\frac{u}{u_{max}-u}\right)^{1/\gamma_{b}}}+ \alpha \frac{m_{a} m_{b}}{\left(\frac{u}{u_{max}-u}\right)^{\left(1/2\gamma_{a}+1/2\gamma_{b}\right)}}  \end{array} $$

Although analytical expressions for *u* can be obtained from Eqs. 15 and 16 only under the restrictions of either a fixed maximum effect *a*_*max*_=*b*_*max*_ and identical slope parameters *γ*_*a*_=*γ*_*b*_ or of different maximum effects and identical slopes of unity *γ*_*a*_=*γ*_*b*_=1, they are the starting points for several response surface models, e.g., Greco’s model from Eq. 16

17 $$\begin{array}{@{}rcl@{}} u_{Greco} & = & u_{max} \frac{\left(m_{a}+ m_{b} + \alpha \times m_{a} m_{b}\right)^{\gamma}}{1 + \left(m_{a}+ m_{b} + \alpha \times m_{a} m_{b}\right)^{\gamma}}  \end{array} $$

or the GCA expression [6] from Eq. 15. It permits different maximum effects but is limited to *γ*=1.

18 $$\begin{array}{@{}rcl@{}} u_{GCA} & = & \frac{a_{max} m_{a}+b_{max} m_{b}}{1 + a_{max} m_{a}+b_{max} m_{b}}  \end{array} $$

Hence, *u*_*Greco*_ (for *α*=0) and *u*_*GCA*_ are special cases of *u*_*Hill*_. The same holds true for Chou and Talalay’s mutually exclusive model [11]. It was derived from the the median effect principle, assuming both a constant *u*_*max*_ and *γ*

19 $$\begin{array}{@{}rcl@{}} u_{{Chou}_{ex}} & = & u_{max} \frac{\left(m_{a}+ m_{b} \right)^{\gamma}}{1 + \left(m_{a} + m_{b} \right)^{\gamma}}  \end{array} $$

Their mutually non-exclusive model [11] is an ad hoc extension of Eq. 19

20 $$\begin{array}{@{}rcl@{}} u_{{Chou}_{nex}} & = & u_{max} \frac{\left(m_{a}+ m_{b} + m_{a} m_{b}\right)^{\gamma}}{1 + \left(m_{a}+ m_{b} + m_{a} m_{b}\right)^{\gamma}}  \end{array} $$

Although it has been criticized by several authors [1, 5, 33] because of its questionable validity, it is one of the most often used models in the literature [34]. For *γ*=1 it becomes the Bliss IA expression (Eq. 13, with *α*=*β*=1 and *u*_*max*_=*a*_*max*_=*b*_*max*_). Chou’s models are related to Greco’s expression for identical slopes *γ*=*γ*_*a*_=*γ*_*b*_ and identical *u*_*max*_, i.e., $\phantom {\dot {i}\!}u_{{Greco}}=u_{{Chou}_{{ex}}}$ for *α*=0 and $\phantom {\dot {i}\!}u_{{Greco}}=u_{{Chou}_{{nex}}}$ for *α*=1.

Minto [30] proposed a model that solved the problem of the different denominators in Eq. 15 by expanding *u*_*max*_ and *γ* in polynomials in a parameter *Θ*. Fidler [28] extended Minto’s approach by adding an interaction term. Their model is

21 $$\begin{array}{@{}rcl@{}} u(\Theta_{p})&=& \frac{u_{max}(\Theta_{p}) \left[m_{a}+m_{b} + \alpha \times f \times \sqrt{m_{a} m_{b}}\right]^{\gamma(\theta_{p})}}{1+\left[m_{a}+m_{b} + \alpha \times f \times \sqrt{m_{a} m_{b}}\right]^{\gamma(\theta_{p})}} \\ \theta_{p} &=& \frac{m_{a}}{m_{a} + m_{b}}  \end{array} $$

where *α* indicates the type of interaction. Minto’s model corresponds to *α*=0, *α*>0 means synergism and *α*<0 antagonism. *f*(*s,w*,*Θ*_*p*_) resembles a generalized *Γ*-distribution, and *u*_*max*_(*Θ*_*p*_) and *γ*(*Θ*_*p*_) are functions of the potency fraction *Θ*_*p*_ and *f*(*s,w*,*Θ*_*p*_). *Θ*_*p*_ ranges from 0 (drug A only) to 1 (drug B only).

Minto’s model differs from the logistic Hill surface only in the functional forms of *u*_*max*_ and *γ*. By truncating their polynomial ansatz for *u*_*max*_ and *γ* after the linear terms in *Θ*_*p*_, we have

$$\begin{array}{@{}rcl@{}}  \gamma(\Theta_{p})& = &\alpha \Theta_{p} + \beta (1-\Theta_{p}) = \gamma_{Hill} \\  u_{max}(\Theta_{p}) & = & a_{max} \Theta_{p} + b_{max} (1-\Theta_{p}) = u_{max} (Hill)  \end{array} $$

Thus by making *γ*(*Θ*_*p*_) and *u*_*max*_(*Θ*_*p*_) symmetric with respect to *m*_*a*_ and *m*_*b*_, *u*_*Minto*_ becomes identical to *u*_*Hill*_. However, inclusion of higher powers of *Θ*_*p*_ leads to violations of the boundary conditions and the sham compliance requirement.

### Examples

As identical theoretical concepts handling mixture effects have been developed by scientists from different disciplines, there is some confusion in the nomenclature used in the literature. We shall use the term ’Bliss independent action’ in the subsequent discussions when referring to the probabilistic independent action ansatz, the exception being examples from agrochemistry, where we use the term ‘Colby’s formula’. This is justified because of its extensive use in the agrochemical literature.

In general, however, one has to keep in mind that a numerical evaluation of a model by comparison with experimental data is difficult because often the error bars of the experiments are large or unknown.

#### Crop protection agents

To demonstrate the applicability of the Hill model old mixture data from the crop protection area were chosen. All pesticides involved achieve an *u*_*max*_ of 100%. For the pairs of atrazine/alachlor [12] (herbicides), aldrin/dieldrin [35] (insecticides), and oxadixyl/mancozeb [36] (fungicides) the root mean square errors of prediction are shown in Table 2. The mixtures are equally well described by using variable slopes or slopes of 1. For all pairs of pesticides the original conclusions are confirmed: neither the herbicide- nor the insecticide-mixtures show deviations from the ’expected’ response, whereas in the case of the fungicide mixtures the differences between expection and experiment indicate the presence of synergism.

**Table 2:** Dependence of agrochemical mixture effect predictions on Hill parameters

	Slope *γ*=1		Slope *γ* variable		
Ligand	*X*_50_^a^	RMSE^b^	*X*_50_^a^	Slope	RMSE^b^
Atrazine	0.057		0.059	0.96	
Alachlor	0.082		0.076	1.45	
Atrazine + Alachlor		9			9
Aldrin	0.008		0.010	2.20	
Dieldrin	0.004		0.005	1.95	
Aldrin + Dieldrin		10			8
Oxadixyl	18		19	1.37	
Mancozeb	294		293	0.99	
Oxadixyl + Mancozeb		25			26

All pesticides are assumed to achieve individual maximum effects of 100%. The magnitude of the deviations of mixture predictions from the experimental data is an indication of synergistic effects

^a^ *LC*_50_ (in kg/ha) for herbicides, *LD*_50_ (in g a.i./100 ml kerosene solution) for insecticides, *EC*_50_ (in mg/l) for fungicides;

^b^root-mean-square error of efficacy prediction for mixture

#### Pyrethroids

**Fig. 1:**
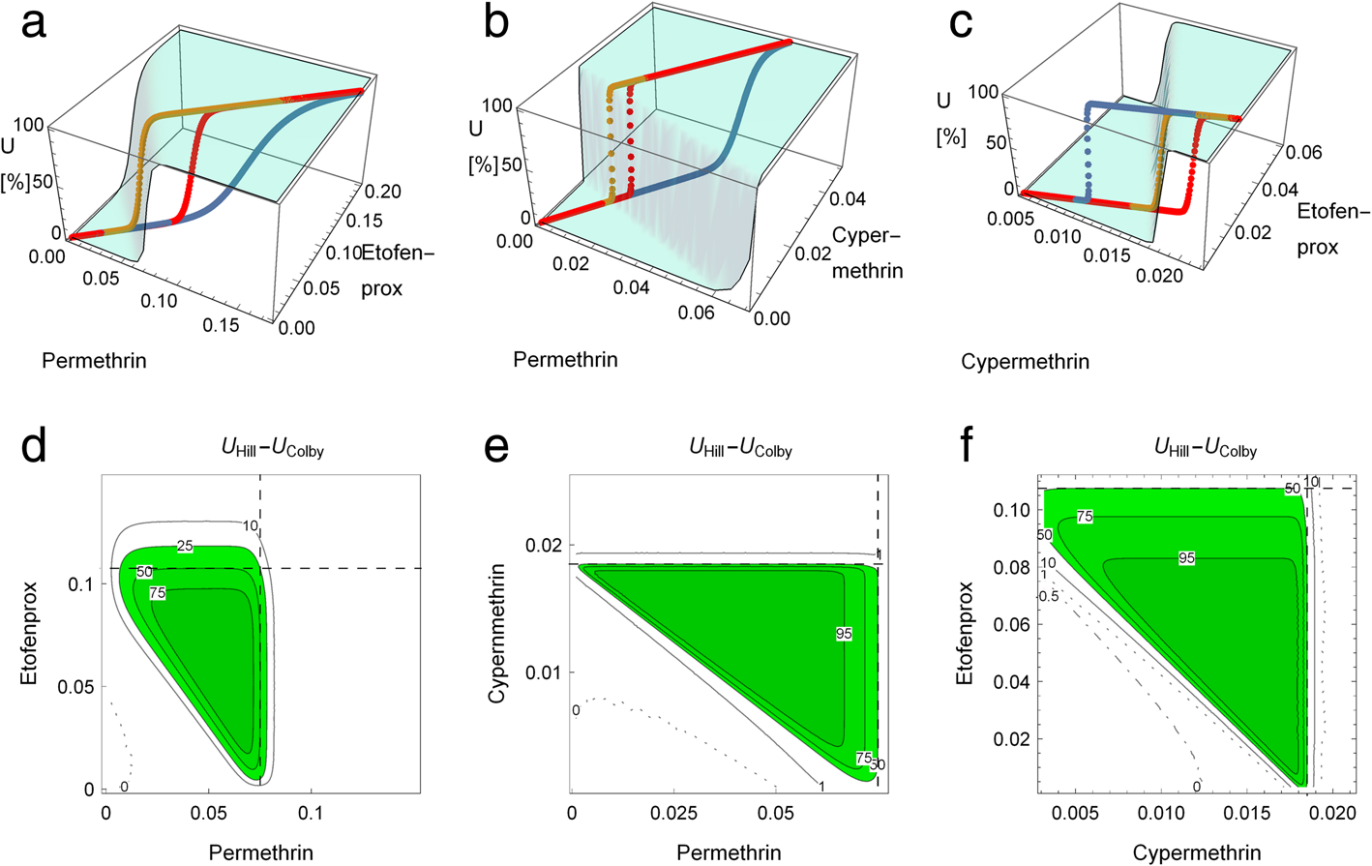
Hill (*green*), Colby (*red*) and experimental (*blue*) mortality curves for pyrethroid mixtures, together with the Hill response surfaces (*cyan*). Mortality in %, doses in *μ* *g*/*cm* ^2^. **a** permethrin + etofenprox (antagonistic) **b** permethrin + cypermethrin (antagonistic) **c** cypermethrin + etofenprox (synergistic); **d**-**f** Contour plots of differences between Hill- and Colby-surfaces (in %) for **a**, **b** and **c**. *Dashed lines* indicate the respective *EC*_50_ values

Mixtures of pyrethroids can act both as synergists and antagonists. While permethrin/etofenprox and permethrin/cypermethrin show antagonism, cypermethrin/etofenprox act synergistically. These effects are only insufficiently described by percentages of 41, 58 and 332% [37] measuring the deviation from additivity. The full picture is given in Fig. 1, where Hill-, Bliss- and experimental dose-responses curves are overlaid with the Hill response surfaces. In this example the differences between the theoretical models are extraordinarily large. The contour plots of *u*_*Hill*_−*u*_*Colby*_ reveal that especially for *x*≤*x*_50_ and *y*≤*y*_50_ Colby’s independent action formula predicts much lower effects than the Hill model. As compared with the latter, the IA approach underestimates antagonism and overestimates synergism. These large differences are caused by the extremely steep dose response curves of permethrin (*λ*=25), etofenprox (*λ*=11) and cypermethrin (*λ*=106), meaning that the effects vary strongly within very narrow dose-ranges.

#### Dioxin-like chemicals

Aryl hydrogen receptor (AhR) ligands were used to compare the toxic equivalence factor (TEF) approach and the more general GCA ansatz by [38] in predicting the expected effect of mixtures containing partial agonists or competitive antagonists. From their supplementary material the Hill curves with variable *γ* were derived and used to predict *u*_*Hill*_. GCA surfaces are Hill-surfaces with slopes of *γ*=1. Hence, one might expect that Hill-surfaces with variable *γ* exhibit slightly improved Mann-Whitney (MW) statistics. However, as the slope parameters from the fits are only marginally different from 1 (c.f. Table 3), the differences between the GCA and Hill in the *E*_*max*_-, *EC*_50_-values and consequently in their response surfaces (not shown) are much smaller than the error bars of the experimental data, while the surface predictions are of comparable quality.

**Table 3:** AhR agonist parameters from GCA- and Hill-fits for mixture predictions

	GCA, *γ*=1 [38]				Hill, *γ* variable		
Ligand	*E*_*max*_(*%*)	*EC*_50_(*M*)	MW^a^	*E*_*max*_(*%*)	*EC*_50_(*M*)	*γ*	MW^a^
TCDF^b^	100	2.9×10^−11^		100	3.2×10^−11^	0.88	
PCB126^c^	99	4.1×10^−10^		100	4.5×10^−10^	0.82	
TCDF + PCB126			0.86				0.86
TCDD^d^	100	7.6×10^−12^		100	6.3×10^−12^	1.29	
PCB105	61	1.4×10^−6^		56	9.2×10^−7^	1.45	
TCDD + PCB105			0.63				0.78
TCDD	100	9.9×10^−12^		100	8.5×10^−12^	1.09	
Galangin	30	4.1×10^−6^		35	4.7×10^−6^	0.79	
TCDD + Galangin			0.79				0.93
TCDD	100	9.1×10^−12^		100	6.5×10^−12^	1.22	
DIM^e^	8	6.6×10^−6^		10	8.5×10^−6^	1.62	
TCDD + DIM			0.65				0.44

Parameters are slopes *γ*, maximum effects *E*_*max*_ and *EC*_50_ values of the agents

^a^MW = Mann-Whitney test for mixture prediction;

^b^2,3,7,8-tetrachlorodibenzofuran;

^c^2,3,3’,4,4’-petachlorobiphenyl;

^d^2,3,7,8-tetrachlorodibenz-p-dioxin;

^e^3,3’-diindolylmethane

### Response surfaces and isoboles

As the classification of mixture effects in terms of interaction indexes is not an adequate means to describe this complex phenomenon, an analysis of responses by looking at the form of iso-effect levels, isoboles, is certainly more appropriate [10, 14, 16]. Deviations from straight lines were used to classify the effects as (Loewe and Bliss) agonistic or antagonistic. From a response surface view [3, 33, 39] isoboles are cuts of the response surface at defined effect levels and the corresponding contour plots are the easiest way to get a full picture for the whole range of dose-combinations. This facilitates the understanding of the fact that linear isoboles are the exception and not the rule and are not a general means of detecting synergism. For critical discussions on the interpretation of the shape of isoboles see, e.g., [5, 40–42].

Here simulated dose-response surfaces are compared, based on two different drugs A and B, both acting according to Hill’s formula. The following parameters were used: Maximum effects *a*_*max*_=0.7, *b*_*max*_=1, median effects $d_{a_{50}}=100$, $d_{b_{50}}=1$, (i.e., *x*_50_=2, *y*_50_=0), and slopes *α*=1, *β*=2.

The comparison will be mainly between *u*_*Hill*_ and *u*_*Bliss*_=*u*_*Colby*_, as the GCA model *u*_*GCA*_ and Chou’s mutually exclusive model $\phantom {\dot {i}\!}u_{{Chou}_{ex}}$ are special cases of the logistic surface *u*_*Hill*_, and under special assumptions Chou’s mutually non-exclusive model $\phantom {\dot {i}\!}u_{{Chou}_{{nex}}}$ reduces to Bliss IA.

**Fig. 2:**
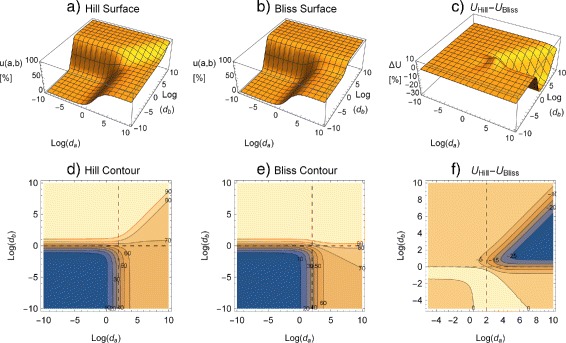
Response surfaces (**a**–**c**) and isoboles (**d**–**f**) for binary mixtures. Effects are given in %. **a**, **d** Hill surface; **b**, **e** Bliss surface; **c**, **f** Difference plots *u*_*Hill*_−*u*_*Bliss*_ show that *u*_*Bliss*_≥*u*_*Hill*_. (Parameters: *u*_*min*_=0, *a*_*max*_=0.7, $d_{a_{50}}=100$, *α*=1, *b*_*max*_=1.0, $d_{b_{50}}=1$, *β*=2). *Dashed lines* denote the respective *d*_50_ values

In Fig. 2 differences between the two surfaces show up at high doses of both mixture partners. There IA would find large synergistic effects of up to 30% where the Hill model would indicate ‘null-interaction’.

**Fig. 3:**
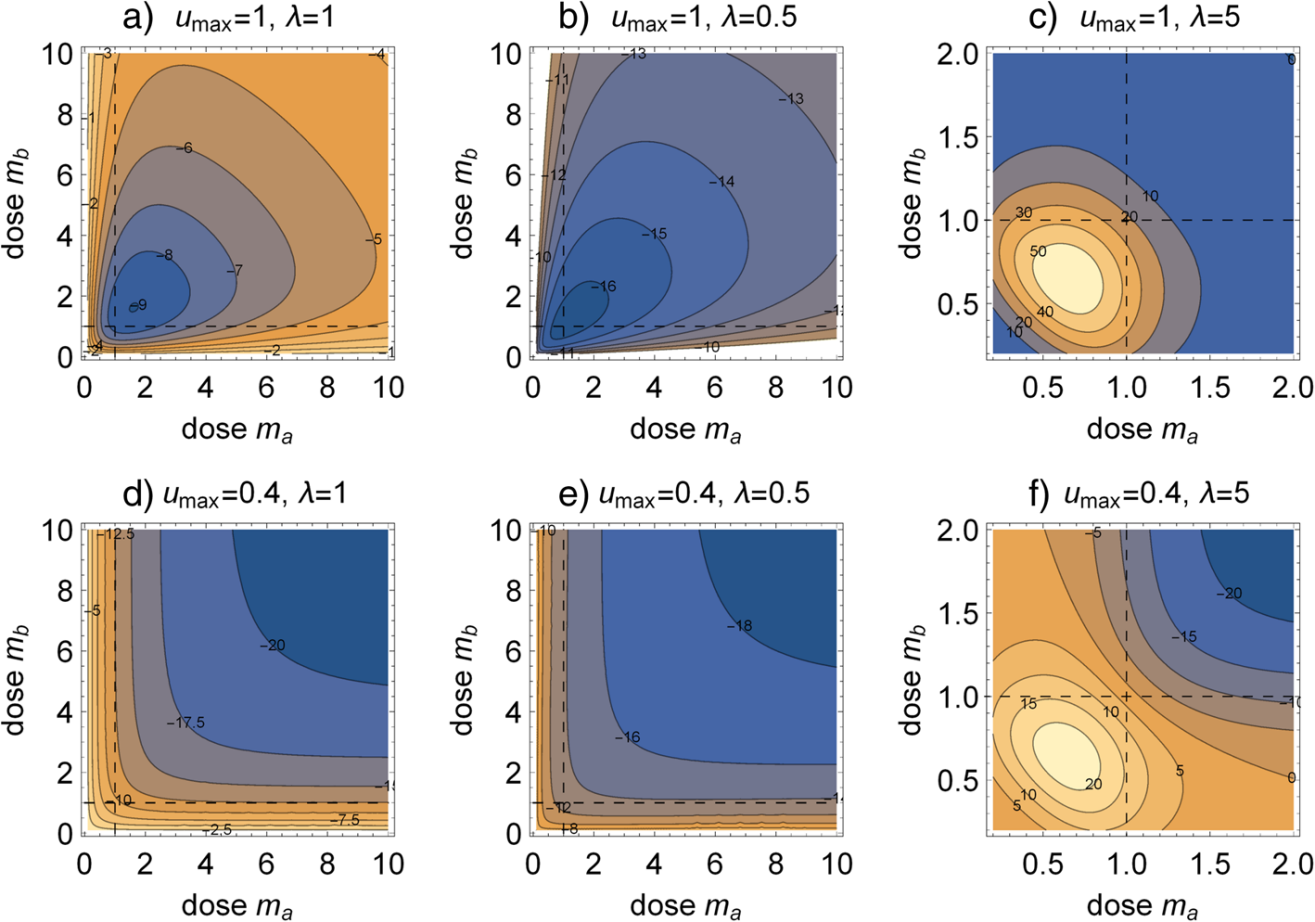
Contour plots of differences *u*_*Hill*_−*u*_*Bliss*_ between Hill- and Bliss-response surfaces (in %). Shown for three slope parameters *λ*=1, 0.5 and 5 for agents A and B. **a** – **c** *u*_*max*_=1, **d** – **f** *u*_*max*_=0.4. *Dashed lines* indicate effects at normalized doses *m*_*x*_=1

Some trends can be observed: For identical maximum effects and slopes, *u*_*Hill*_−*u*_*Bliss*_ is positive at doses below *d*_50_ and increases with increasing *u*_*max*_ and *λ*, and it is negative else, almost independent of the dose range. This means that Bliss independent action tends to overestimate synergism at doses smaller than their median effect doses and to underestimate synergism at doses above. At low doses the size of the difference increases with *u*_*max*_ and *λ*. If the maximum effects differ, the effect differences at higher doses increase with increasing *a*_*max*_−*b*_*max*_. Some of these findings are illustrated in Fig. 3 for binary mixtures of agents with identical parameters: maximum effects (*u*_*max*_=1 and *u*_*max*_=0.4) were combined with three different slope parameters (*λ*=0.5,1.0,5.0). Using scaled doses *m*=*d*/*d*_50_ for the axes simplifies the picture without obscuring the essentials.

### Iso-surfaces

**Fig. 4:**
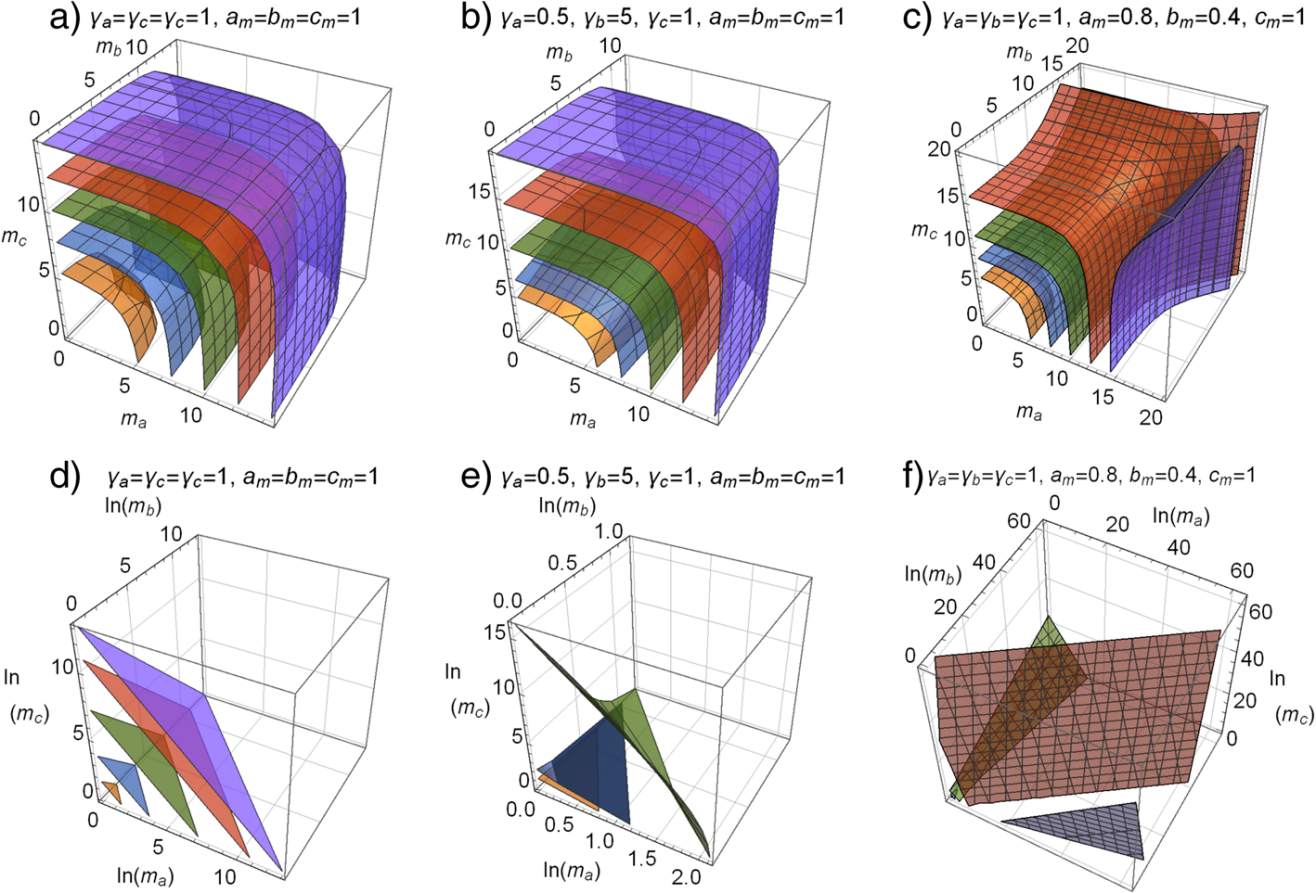
Iso-surfaces for ternary mixtures at 10, 25, 50, 75 and 90% effect levels. Shown on logarithmic (**a**–**c**) and linear (**d**–**f**) dose scales. **a**, **d** sham combination, *α*=1,*u*_*max*_=1; **b**, **e** Mixture with variable slopes, *α*=0.5,*β*=5,*γ*=1 and *u*_*max*_=1; **c**, **f** Mixture with variable maximum effects, *a*_*max*_=0.8, *b*_*max*_=0.4, *c*_*max*_=1 and slope *λ*=1

For ternary mixtures the pendants of isoboles are iso-surfaces, i.e., projections of the four-dimensional response surfaces to three-dimensional ones defined by triples of doses leading to a specified response. A visualization of effects resulting from more than three agents in one graphical object is hardly possible. As shown for some fictitious ternary mixtures in Fig. 4, planarity is achieved only if all mixture components have identical slope parameters. In this case different maximum effects of the mixture partners do not affect planarity. Iso-surfaces of mixtures of agents having different slopes are non-planar.

**Fig. 5:**
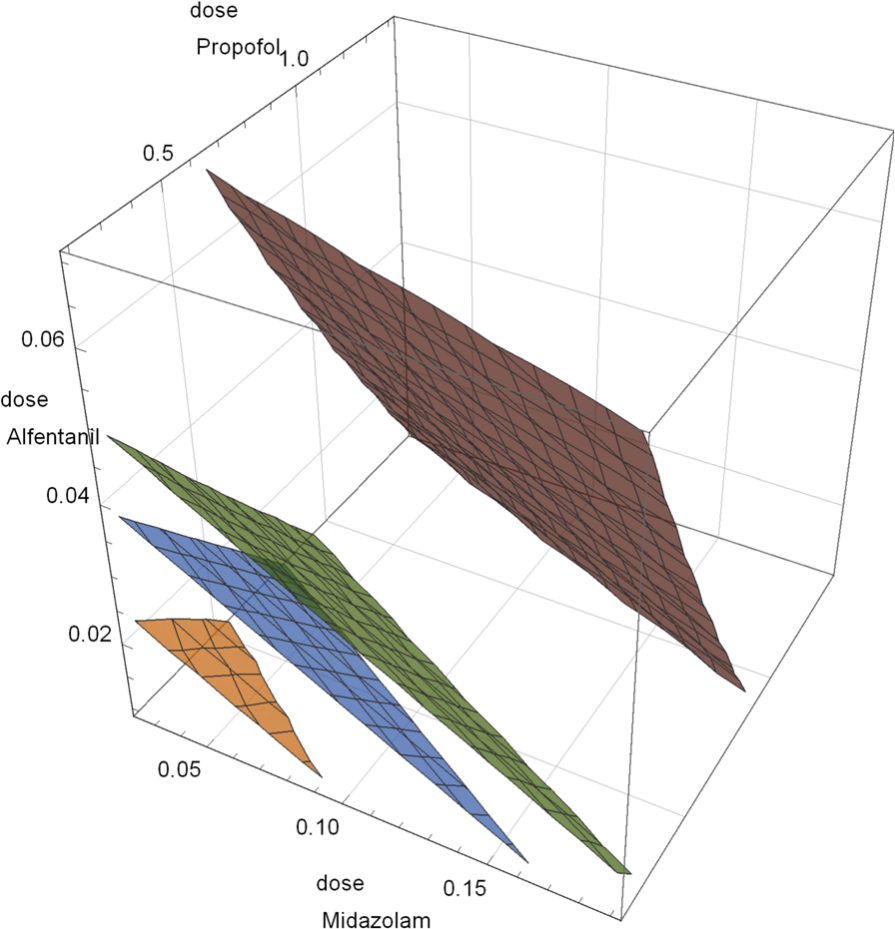
Iso-surfaces (10, 50, 75, and 99%) for ternary mixtures of midazolam, propofol, and alfentanil. Doses in [mg/kg]. Deviation from planarity results from differing slopes of the individual anesthetics

This is shown for ternary anesthetic mixtures [43] as analyzed in great detail by [28] and [30]. For the present purpose the data for midazolam, propofol, and alfentanil were fitted to Hill dose-response curves assuming effect ranges from 0 (no hypnosis) to 1 (full hypnosis). The characteristics of fits and of predictions for the mixtures are summarized in Table 4. The corresponding iso-surfaces in Fig. 5 show slight deviations from planarity which result from different slopes (4.8–11.1) of the pure agents. As the Hill model provides only null-interaction surfaces, the size of the deviations of experimental data from the Hill prediction may give some hints on the presence of synergy. While the RMSEs of fit for the pure compounds are of the order of 5%, those for mixture prediction are of the order of 40%, indicating the presence synergistic effects of increasing magnitude for the binary mixtures of propofol/alfentanil, midazolam/propofol and midazolam/alfentanil, while the ternary mixture is comparable to the best binary one.

**Table 4:** Fit parameters for anesthetics and their mixtures

Drug	*D*_50_(*mg*/*kg*)	Slope	RMSE^a^
Midazolam	0.144	4.83	0.054
Propofol	1.078	11.14	0.035
Alfentanil	0.093	5.69	0.069
Midazolam/propofol			0.387
Midazolam/alfentanil			0.477
Propofol/alfentanil			0.228
Ternary mixture			0.461

^a^RMSE = root-mean-square error with respect to exptl. data. For pure compounds RMSE refers to fits, for mixtures it refers to predictions

## Conclusions

Starting from a logistic PDE, analytical expressions for the response surfaces of n-component mixtures have been derived under the sole provision that each a.i. is described by a sigmoid dose-response curve. No further assumptions are required. The resulting ‘null-interaction’ surfaces, i.e., Hill-surfaces in the absence of of synergistic or antagonistic effects, provide the ‘expected’ response for each dose combination. Deviations from this reference response in order to quantify synergism or antagonism should possibly be handled by some sort of perturbation theory.

The Hill approach provides a framework to classify several models describing mixtures like ADM (Loewe CA, GCA), MSM (Colby, Bliss, Finney, Abbott) or Chou’s ‘unified general theory’ and various response surface models. It can be applied to mixtures of compounds having different maximum effects and differing slope-parameters. Many Loewe-additivity based approaches are found to be special cases of the Hill surface while Bliss IA is incompatible with the logistic ansatz.

The independent action model is frequently used, e.g., in patent applications to quantify synergistic effects [44]. Its outcome should be checked by comparison with the Hill response surface for any relevant mixture ratio. From the literature examples discussed two scenarios can be distinguished: For slopes of *γ*≈1 the response surfaces calculated from MSM formulae, deviate from the Hill surface by predicting synergism where the Hill model indicates ‘null-interaction’, especially at doses below the *d*_50_-values of the components. In fact, under these conditions synergism postulated by MSM is an upper boundary for the synergism predicted by the Hill surface approach. However, if both mixture partners alone can cause effects of 100% or if the differences between their slopes are small either the deviations or the dose ranges affected are small.

For *γ*≫1 the differences between Hill- and MSM-surfaces become extremely large, but they are restricted to those small dose-ranges where the effects change rapidly.

As a consequence for claiming synergistic effects at doses below the respective *d*_50_-values, special caution is required whenever the predictions from MSM and Hill approaches differ considerably.

An in-depth analysis of real mixtures would have to take into account not only errors in the models but also other sources of errors like the error statistics of experimental data.

## Abbreviations

ADM: Additive dose model
AhR: Aryl hydrogen receptor
CA: Concentration addition
*EC*_50_: Effective concentration 50
GCA: Generalized Concentration addition
IA: Independent action
*LC*_50_: Lethal concentration 50
*LD*_50_: Lethal dose 50
MSM: Multiplicative survival model
MW: nonparametric Mann-Whitney U test
NOEL: No-effect level
ODE: Ordinary differential equation
PDE: Patial differential equation
RMSE: Root-mean-square error
TEF: Toxic equivalence factor
*u*_*Bliss*_: Bliss independent action response
$\phantom {\dot {i}\!}u_{{Chou}_{nex}}$: Chou’s mutually non-exclusive response
$\phantom {\dot {i}\!}u_{{Chou}_{ex}}$: Chou’s mutually exclusive response
*u*_*Colby*_: Bliss independent action response
*u*_*Greco*_: Loewe additivity based response
*u*_*GCA*_: Generalized concentration addition response
*u*_*Hill*_: Hill response surface
*u*(*Θ*_*p*_): Minto’s and Fidler’s polynomial response
